# Metabolic Reprogramming of Castration-Resistant Prostate Cancer Cells as a Response to Chemotherapy

**DOI:** 10.3390/metabo13010065

**Published:** 2022-12-31

**Authors:** Greta Petrella, Francesca Corsi, Giorgia Ciufolini, Sveva Germini, Francesco Capradossi, Andrea Pelliccia, Francesco Torino, Lina Ghibelli, Daniel Oscar Cicero

**Affiliations:** 1 Dipartimento di Scienze e Tecnologie Chimiche, Università di Roma “Tor Vergata”, 00133 Rome, Italy; 2 Dipartimento di Biologia, Università di Roma “Tor Vergata”, 00133 Rome, Italy; 3 Dipartimento di Medicina dei Sistemi, Oncologia Medica, Università di Roma “Tor Vergata”, 00133 Rome, Italy

**Keywords:** exometabolomics, nuclear magnetic resonance, cellular metabolism, prostate cancer, etoposide, phoenix rising, chemoresistance

## Abstract

Prostate cancer at the castration-resistant stage (CRPC) is a leading cause of death among men due to resistance to anticancer treatments, including chemotherapy. We set up an in vitro model of therapy-induced cancer repopulation and acquired cell resistance (CRAC) on etoposide-treated CRPC PC3 cells, witnessing therapy-induced epithelial-to-mesenchymal-transition (EMT) and chemoresistance among repopulating cells. Here, we explore the metabolic changes leading to chemo-induced CRAC, measuring the exchange rates cell/culture medium of 36 metabolites via Nuclear Magnetic Resonance spectroscopy. We studied the evolution of PC3 metabolism throughout recovery from etoposide, encompassing the degenerative, quiescent, and repopulating phases. We found that glycolysis is immediately shut off by etoposide, gradually recovering together with induction of EMT and repopulation. Instead, OXPHOS, already high in untreated PC3, is boosted by etoposide to decline afterward, though stably maintaining values higher than control. Notably, high levels of EMT, crucial in the acquisition of chemoresistance, coincide with a strong acceleration of metabolism, especially in the exchange of principal nutrients and their end products. These results provide novel information on the energy metabolism of cancer cells repopulating from cytotoxic drug treatment, paving the way for uncovering metabolic vulnerabilities to be possibly pharmacologically targeted and providing novel clinical options for CRPC.

## 1. Introduction

Prostate cancer (PC) is the second most common cancer in men, accounting for 7% of newly diagnosed cancers in men globally (15% in developed countries) [1]. At diagnosis, PC is mainly a hormone-sensitive disease (HSPC), and androgen deprivation therapy (ADT), along with local treatment (i.e., radiotherapy and/or radical prostatectomy), may provide high rates of long-term disease control and remission based on the stage of the disease. However, this almost invariably turns into aggressive castration-resistant PC (CRPC), requiring a palliative therapeutic strategy, including cytotoxic agents [1].

Tumor recurrence after chemotherapy implies the incomplete killing of tumor cells (minimal residual disease). Surviving cells may repopulate via the “Phoenix Rising” pathway, where apoptotic cells themselves emit proliferative signals aimed at repopulation and chemoresistance [2]. It is emerging that such processes are mediated by epithelial-to-mesenchymal transition (EMT), an epigenetic-based signaling pathway forced by therapy-induced damage, which increases cancer cells’ migratory capability, promoting the establishment of metastases [3].

Etoposide, a widely used anticancer drug [4], is a poison of topoisomerase II. This ubiquitous enzyme regulates DNA replication and transcription by relaxing the conformational stress caused by DNA and RNA polymerases advancement that distorts the double helix by cleaving the sugar-phosphate chain of one DNA strand, bypassing the DNA strand overlap, and finally re-ligating the sugar-phosphate bond. Etoposide forms a covalent complex between topoisomerase II and the cleaved DNA (“cleavable complex”), inhibiting re-ligation and resulting in the accumulation of DNA breaks, which can trigger the apoptotic processes. Etoposide phosphate, also known as VP-16, has been included in the National Comprehensive Cancer Network guidelines for the treatment of mCRPC since 1997 [5] and is now used as a co-treatment with platinum agents for the treatment of aggressive forms of neuroendocrine PC (NEPC) [6].

We have recently established an in vitro model of cancer repopulation and acquired cell resistance (CRAC) induced by etoposide on PC3 CRPC cells with neuroendocrine traits, showing that cells that survive the treatment repopulate via EMT and Phoenix Rising [7]. In this one-pot in vitro model, cells were allowed to grow and adapt to the effects of etoposide without ever being detached from the culture medium. This strategy made it possible to reproduce what happens in vivo upon chemotherapy as accurately as possible. Indeed, after a phase of apoptosis, surviving PC3 cells set up a coordinated response to cope with the damage, recapitulating what occurs in tissues. In this process, they undergo epigenetic reprogramming and EMT to repopulate via Phoenix Rising, gaining chemoresistance and undergoing disease progression, thus mimicking post-remission relapse occurring in patients [7].

It is well known that the process of tumor progression implies significant changes in metabolism [8]. It is equally well known that patients’ metabolic state influences the effects and efficacies of chemotherapies [9]. However, little is known about how chemotherapy may affect aberrant cancer metabolism. It would be of paramount importance to understand at a molecular level the metabolism of cancer cells during tumor progression induced by chemotherapy because it may help discover vulnerabilities that may inspire the development of more efficient treatments in preventing, reducing, or at least delaying post-remission relapse in responding patients.

We used an exometabolomics approach to explore how PC3 metabolism may change during etoposide-induced CRAC. We measured cells’ metabolic exchange rates (ERs) with the culture medium through Nuclear Magnetic Resonance (NMR). This way, a time-resolved view of the metabolic pathways exploited by cells to secure nutrients and energy is obtained. This allowed us to establish an omics point of view on the metabolic alterations occurring during recovery from etoposide treatment in PC3 cells, clearly distinguishing the degenerative and quiescent phase from the proliferating conditions, but also indicating that cells repopulating after therapy display metabolic differences with respect to the untreated, suggesting a stable/metastable therapy-dependent metabolic shift.

## 2. Materials and Methods

### 2.1. Cell Culture

Human prostate cancer PC3 cells, derived from bone metastasis of a grade IV prostatic adenocarcinoma, were grown in RPMI 1640 medium supplemented with 10% fetal bovine serum (FBS), 100,000 units/L penicillin, 50 mg/L streptomycin and 200 mM glutamine (Euroclone, Milan, Italy). All cells were grown at 37 °C in a humidified atmosphere of 5% CO_2_ in the air and routinely split by trypsinization with Trypsin-EDTA (Euroclone). Experiments were performed with base viability >98%.

The protocol used in this study followed CRAC’s design as previously described [7].

### 2.2. Vimentin Staining and Analysis

Samples grown over Nunc Lab-Tek chambers (Thermo Fisher, Waltham, MA, USA) were washed with PBS, fixed with 4% paraformaldehyde for 15 min, washed three times with PBS, permeabilized in PBS 0.3% Triton for 10 min, and blocked with 1% BSA for 30 min. The samples were then incubated with primary antibody against vimentin (Sigma-Aldrich, St. Louis, MO, USA) in PBS 1% BSA for 1 h at room temperature. After three washes with PBS, the samples were incubated with the FITC-conjugated secondary antibody (Sigma-Aldrich, St. Louis, MO, USA) at room temperature for 1 h. The samples were washed three times with PBS, and the cell nuclei were finally stained with DAPI (2 µg/mL). Images were captured using a ZEISS Axio Observer microscope; unbiased staining intensity was estimated after background signal elimination through pixel fluorescence analysis according to Carl Zeiss Microscopy GmbH’s ZEN software (version 3.0, Jena, Germany). At least six images for each sample were analyzed to obtain the relative frequency distribution of vimentin fluorescence intensities. Statistical significance between the distributions was calculated via the Mann–Whitney test.

### 2.3. Samples for NMR Analysis

Each cell culture medium was collected, centrifuged to remove cell debris, and immediately frozen at −80 °C. Before preparation for NMR analysis, each sample was thawed at room temperature. In order to remove proteins from the Fetal Bovine Serum (FBS), which was added to the medium to ensure proper cell growth, the sample was subjected to a deproteinization process by ultrafiltration. Then, 500 µL of the sample was inserted in an Amicon Ultra 0.5 filter membrane with a cut-off equal to 3 KDa (previously cleared of glycerol) and subjected to centrifugation at 13,800× *g* at 4 °C for 90 min. Subsequently, 400 µL of the filtrate was placed into a new Eppendorf and added to 200 µL of phosphate buffer (250 mM KH_2_PO_4_/K_2_HPO_4_, pH 7.4) containing 0.467 mM 3-(trimethylsilyl)-2,2,3,3-tetradeutero-propionic acid (TSP), 10% D_2_O and 2% NaN_3_. The final solution was transferred to a 5 mm NMR tube.

### 2.4. ^1^H-NMR Spectroscopy

Acquisition of ^1^H-NMR spectra was performed using a Bruker Avance 700 MHz spectrometer equipped with SampleXpress Lite autosampler and Topspin software (version 2.1, Bruker GmbH, Karlsruhe, Germany); acquisition modes included single-sample experiments and macro experiments (semi-automated mode). For each sample, a ^1^H-NMR spectrum was acquired with a noesypr1d sequence at 298 K, 15.9 ppm spectral window, 1024 scans, 4 dummy scans, 2-s relaxation delay, and 3-s acquisition time.

### 2.5. Spectral Deconvolution

All the spectra were processed using 0.5 Hz of line-broadening followed by manual phase and baseline correction. Chenomx NMRSuite 8.5 (Chenomx Inc., Edmonton, AB, Canada) was used to quantify the metabolites. The spectra database in this software allows a manual deconvolution of the different signals and determines the concentration of the compounds that form the mixture. TSP was set as an internal standard, and 36 metabolites were quantified in almost all samples.

### 2.6. Measurement of the Metabolite Exchange Rates

We have calculated the consumption or excretion of all quantified metabolites, $\Delta {\left[C\right]}_{i}^{k}$, for all five cellular phases by using the following equation:

(1) $$\Delta {\left[m\right]}_{i}^{k}=({\left[C\right]}_{i}^{k}-{\left[C\right]}_{i}^{0})V$$

where ${\left[C\right]}_{i}^{k}$ refers to the concentration of the *i*-metabolite in the *k* phase. The concentration of the reference medium is ${\left[C\right]}_{i}^{0}$ and represents RPMI cultured in the same condition but without the cells.

The concentration differences are then converted into mass differences, $\Delta {\left[m\right]}_{i}^{k}$, taking into account the total volume of the medium.

For each phase, we estimated the growth rate ($k_{p}$) obtained from fitting the *ln(*$n_{cell}$*)* vs. time (t), where $n_{cell}$ is the cell count

(2) $$n_{cell}\left(t\right)=n_{cell}\left(0\right)e^{k_{p}t}$$

This first-order exponential was used to explain the variation in cell number in all phases. Using the cell counts at two different times in the three independent experiments, we estimated the values of $k_{p}\;\mathrm{for}\;\mathrm{each}\;\mathrm{phase}$. For the calculation of $k_{p}$ of untreated cells, they were counted at the end of the 9th day, and $k_{p}$ was calculated using the initial value at time 0. All the results are given in the Supplementary Material (Table S1).

Using this equation, we calculated the area ($A_{p}$) under the curve by integration between the starting (*t*_1_) and final (*t*_2_) times of each phase:

(3) $$A_{p}=\int_{t_{1}}^{t_{2}}n_{cell}\left(0\right)e^{k_{p}t}\;dt=\frac{n_{cell}\left(0\right)}{k_{p}}\left(e^{k_{p}t_{2}}-e^{k_{p}t_{1}}\right)$$

Metabolite exchange rates (*ER*) are calculated using the following equation

(4) $$ER_{i}^{k}=\frac{\Delta {\left[m\right]}_{i}^{k}}{A_{p}}$$

#### Scaling of the Variation Matrix to Obtain a Heatmap

In order to visualize at the same scale, the metabolite variations in the heatmap, the multivariate analysis, and the plots, the (*n*) were converted on a scale between 0 and 1 (for excretions), −1 and 0 (for consumptions), and −1 to 1 (for mixed behaviors), applying the following formulas:

Excretions:

(5) $$\frac{n-min}{max-min}$$

Consumptions:

(6) $$\frac{n-max}{min-max}$$

Mixed:

(7) $$\frac{n}{max\left(\left|min\right|,max\right)}$$

### 2.7. Data Analysis

The heatmap was generated using the R- “heatmaply” package (R version 4.1.3) [10]. The multivariate analysis of the data was carried out using SIMCA-P (version 15.0.2. Umetrics AB, Umea, Sweden). The principal component analysis (PCA) variables were the scaled exchange rates. Univariate statistical analysis was performed using Excel, evaluating *p*-values < 0.05 as significant.

## 3. Results

This study aims to determine the consequences of treatment with the chemotherapy agent etoposide on the metabolism of PC3 cells. To this end, we reproduced our in vitro CRAC model [7] to monitor variations in the metabolic exchange rates (*ERs*) with the external medium of PC3 cells during the different phases they experienced. In this “one-pot” approach, the treated cells function as producers and receptacles of paracrine secretions for subsequent steps [8], mimicking the in vivo situation as accurately as possible.

### 3.1. The CRAC Experiment to Study the Cell Metabolic Changes

PC3 cells were plated in a 5-mL flask; after 72 h post-seeding, they were treated with etoposide for 24 h, washed out, and allowed to recover for 19 days. The medium was changed every 48 or 72 h to warrant an adequate supply of the main nutrients, such as glucose and glutamine.

**Figure 1:**
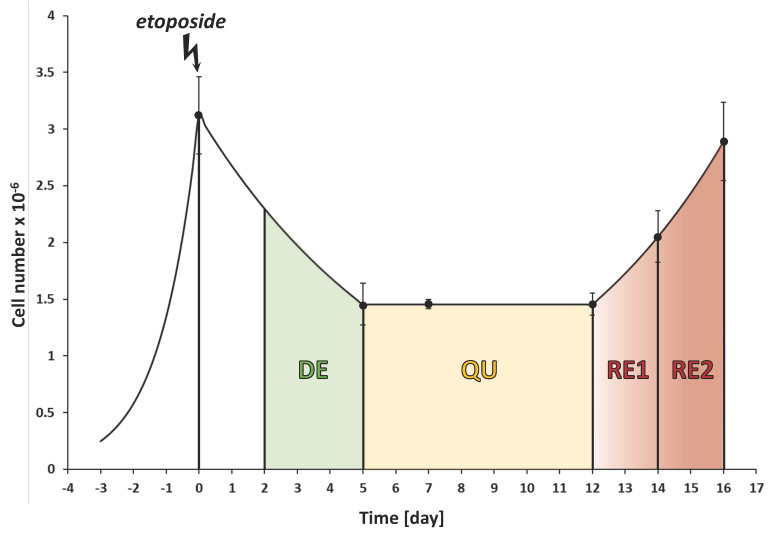
Evolution of the culture after etoposide treatment: DE, QU, RE1, and RE2 represent the degenerative, quiescent, and repopulation phases. Vertical lines indicate medium changes; the colored areas show the successive stages of CRAC. Each dot represents the time when cells were counted, and the bar is the corresponding standard deviation of its value.

Figure 1 shows the cell number variation as a time function. Etoposide treatment induced an initial degenerative phase (DE) in which the number of cells strongly decreased. The surviving cells transited through a seven-day quiescent phase (QU), when cells assumed novel and different morphologies, though their number remained constant. These cells express high levels of the mesenchymal marker vimentin, indicating the occurrence of EMT [7]. After 12 days from the chemotherapeutic insult, cell number began increasing due to repopulation via Phoenix Rising. Because the EMT characteristics acquired in the quiescent phase are present at the beginning of repopulation, though slowly decreasing, this phase was divided into two sub-phases (RE1 and RE2). The control sample (CTR) consists of cells plated at low density and allowed to grow for ten days in the same flask (i.e., without trypsinization), refreshing the medium every 48/72 h. This experiment was conducted in triplicate.

### 3.2. ^1^H NMR Spectroscopy Provided Metabolite Concentrations in Culture Medium Samples

**Figure 2:**
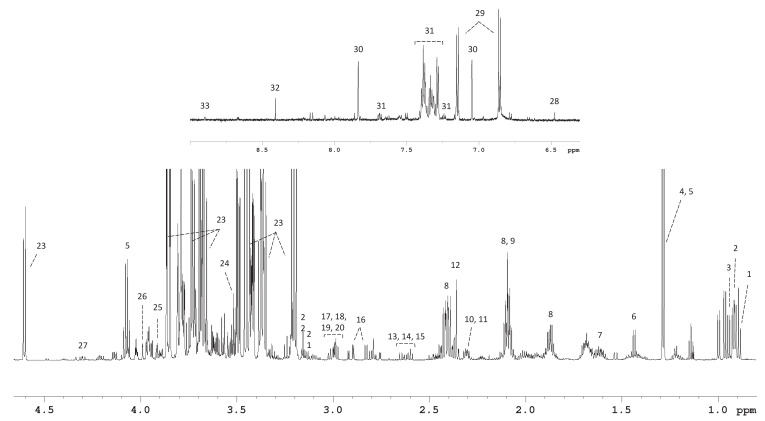
A ^1^H-NMR spectrum of an RPMI medium sample. Metabolites: 1, isoleucine; 2, leucine; 3, valine; 4, threonine; 5, lactate; 6, alanine; 7, arginine; 8, glutamine; 9, methionine; 10, glutamate; 11, proline; 12, succinate; 13, methionine; 14, citrate; 15, aspartate; 16, asparagine; 17, lysine; 18, creatine; 19, creatinine; 20, ornithine; 21, cystine; 22, choline; 23, glucose; 24, glycine; 25, serine; 26, fructose; 27, trans-4-Hydroxy-L-proline; 28, fumarate; 29, tyrosine; 30, histidine; 31 tryptophan; 31, phenylalanine; 32, formate; 33, nicotinurate.

We measured the metabolic consumption and excretion profiles of all CRAC’s phases using ^1^H-NMR spectroscopy. Figure 2 shows a typical NMR spectrum recorded at 700 MHz of a medium sample. After deconvolution, we followed the concentrations of 36 compounds, including twenty-two amino acids, nine organic acids, glucose, choline, o-acetylcholine, creatine, and creatinine. Through this analysis, all metabolite concentrations could be determined in a single experiment without needing a chromatographic separation.

Metabolite concentrations of each sample were subtracted from those from a medium held under the same conditions but devoid of cells to quantify changes in consumption and excretion (Table S2). Only significant differences were considered, whereas those differences showing *p* > 0.05 were assumed to be zero.

### 3.3. Etoposide Exposure Alters PC3 Metabolic Exchange Rates

The exometabolomics approach can provide a time-resolved view of which metabolic pathways are exploited by cells to secure nutrients and energy. This information is contained in the *ERs* for each cellular phase ($\mathrm{pmol}\cdot {\mathrm{cell}}^{-1}\cdot {\mathrm{day}}^{-1}$), which were obtained by multiplying the concentration differences by the total volume of the medium and dividing the result by the area under the growth curve. The signs of these *ERs* show that 20 metabolites were consumed and 15 excreted, while the glycine usage was mixed. The consumption profile is dominated by glucose, glutamine, and branched-chain amino acids (BCAAs) since they account for 91% of all the carbon atoms incorporated by the cells: glucose alone provides 75%, while Glutamine, BCAAs, and arginine constitute the main nitrogen source, accounting for 76% of the total (Table S3).

**Figure 3:**
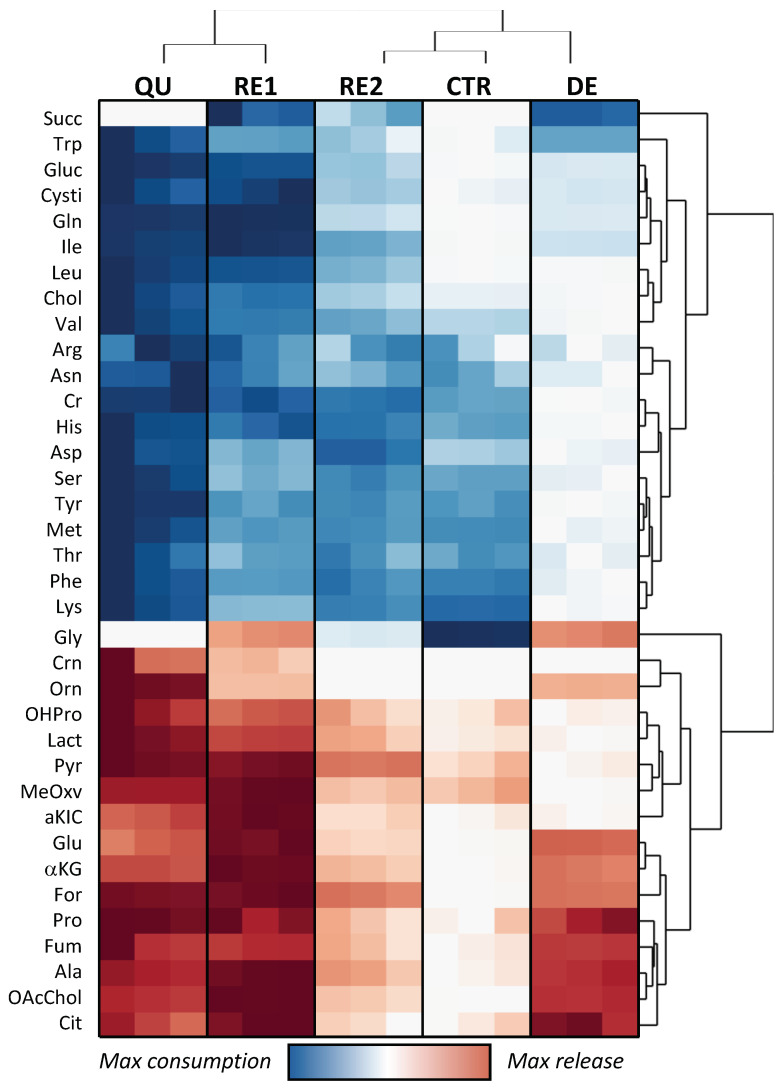
Heatmap of the consumption and excretion rates of control PC3 and during the successive phases of CRAC. Abbreviations used: for: formate, Fum: fumarate, OAcChol: O-acetylcholine, Cit: citrate, αKG: α-ketoglutarate, αKIC: α-ketoisocaproate, MeOxV: 3-methyl2-oxovalerate, Pyr: pyruvate, Lact: lactate, OHPro: 4-hydroxyproline, Crn: creatinine, Cr: creatine, Chol: choline, Gluc: Glucose, Cysti: cysteine, Succ: succinate, amino acids are abbreviated using the three-letter code.

A heatmap with the results on CRAC’s exometabolomic fluxes, scaled in each line between their minimum and maximum values, is reported in Figure 3. This unsupervised cluster analysis hierarchically classifies the cells’ stages through the relationship between consumption and excretion rates. As a result, QU and RE1 show a higher global exchange of metabolites with respect to CTR, RE2, and DE. On the other hand, the CTR and DE profiles are practically opposite, indicating the vast impact that etoposide treatment exerts on PC3 metabolism.

### 3.4. Omics Correlations Reveal Different Metabolic Regulations during Successive CRAC Phases

Through a principal component analysis (PCA) of the metabolic *ERs*, it was possible to extract the maximum information in the complete set of metabolites—the omics information. This way, the biochemical relationship between these molecules that regulate cellular metabolic fluxes can be revealed. Three indicators were also considered in addition to the exchange rates: (i) the lactate/glucose ratio, which measures the number of lactate molecules formed from each glucose molecule consumed and, thus, the efficiency of aerobic glycolysis; (ii) the alanine/glucose ratio, which measures the conversion degree of glucose in alanine via pyruvate and can be related to the OXPHOS activity [11], and (iii) the rate of cell growth, expressed with the exponential value of the growth constant that represents the variation factor in the number of cells in one day. These indicators allowed us to identify which metabolic fluxes correlate with the glycolysis/OXPHOS balance or cellular growth rate.

**Figure 4:**
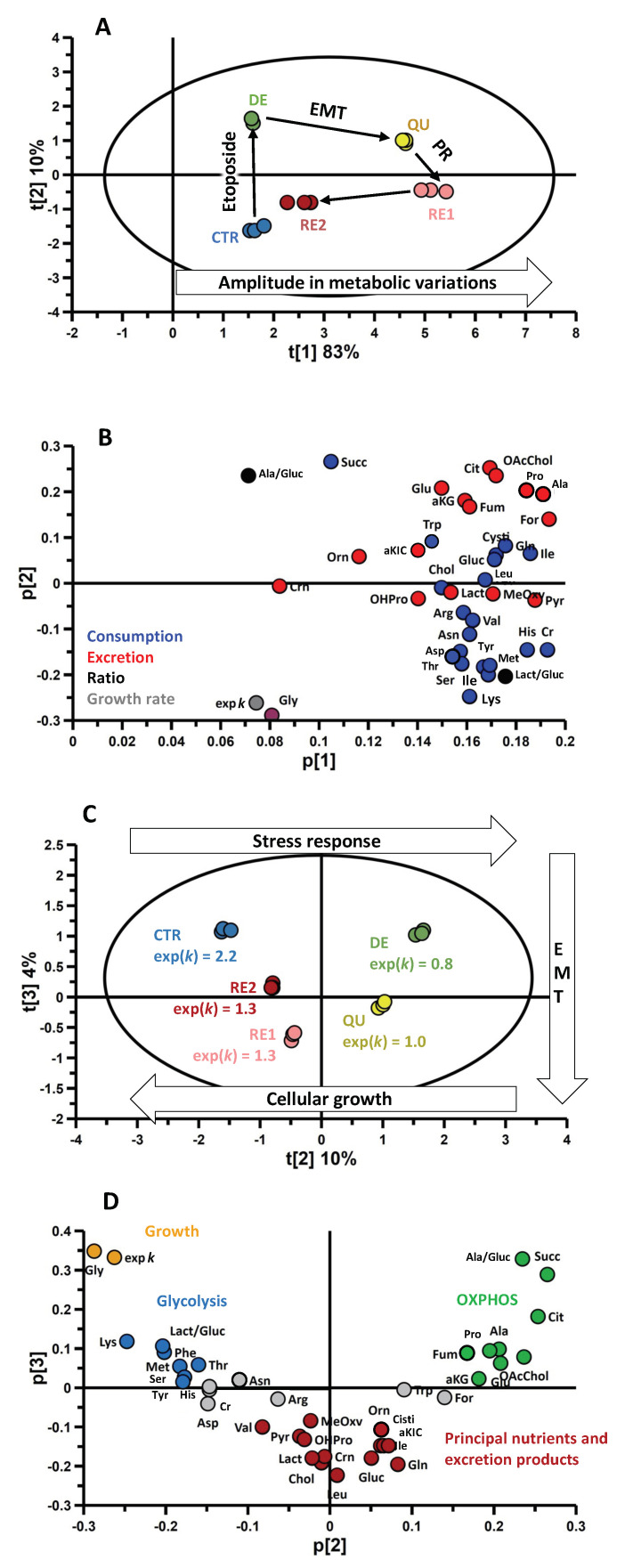
Principal component analysis (PCA)’s score plots of t[1] vs. t[2] (A) and t[2] vs. t[3] (**C**) calculated with the cellular *ERs* and the metabolic indicators (exp(*k*), lactate/glucose, and alanine/glucose). The corresponding loading plots of (**A**) and (**C**) are (**B**) and (**D**), respectively. In the loading plot (**B**), colors refer to consumption (blue), excretion (red), ratios (black), and exp(*k*) (gray). In the loading plot (**D**), orange, blue, red, and green metabolites are related to cellular growth, glycolysis, EMT, and OXPHOS activity, respectively. Abbreviations used are described in Figure 3.

Using the PCA approach, it was possible to reduce a 39-dimension problem to just three principal components, which explains 97% of the total data variance (Figure 4). The first principal component, which accounts for 83% of the total data variance, discriminates QU and RE1 metabolic profiles from the other phases (Figure 4A) based on the fast *ERs* of almost all metabolites (Figure 4B). This result can be interpreted as the consequence of faster metabolic activity during QU and RE1, as already discussed in the analysis of the heatmap of Figure 3.

The second and third components (Figure 4C) are orthogonal to the first, indicating that they are not influenced by the higher global exchange observed during RE1 and QU. For this reason, they reflect how the metabolic exchange profiles of the various phases change in composition (Figure 4D).

**Figure 5:**
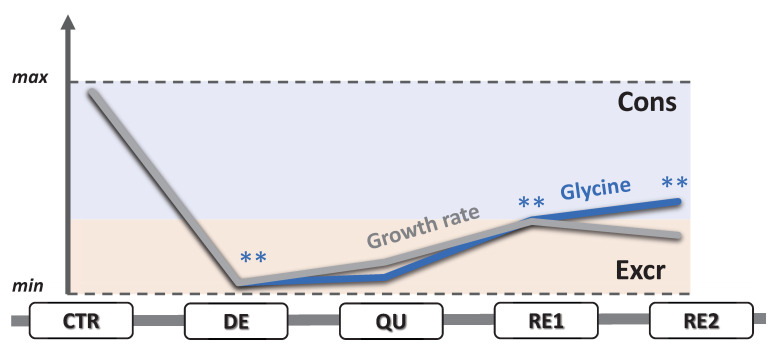
Variation of the glycine exchange and growth rates scaled between their minimum and maximum values. Within the blue and orange areas fall the phases that consume (Cons) or excrete (Excr) glycine. Significant differences with respect to the previous phase are indicated with ** for *p* < 0.01.

Two effects coexist in the second component, which explains 10% of the total variance: the metabolic change caused by etoposide treatment and its consequence on cell growth rate. The latter strongly correlates with glycine *ERs* (Figure 4D and Figure 5). This amino acid was consumed by dividing cells (CTR, RE1, and RE2) and excreted by nondividing cells (DE, QU).

On the second component, the weight of the lactate/glucose and alanine/glucose ratios is opposite. This indicates that cells in phase RE2 and CTR showed high glycolytic activity that correlates with increased consumption of essential (lysine, methionine, phenylalanine, and threonine) and nonessential (serine and tyrosine) amino acids. On the other hand, cells in the DE and QU phases showed high OXPHOS activity corroborated by a high exchange rate of tricarboxylic acid (TCA) cycle intermediates (fumarate, succinate, citrate, and α-ketoglutarate).

### 3.5. EMT Correlates with Higher Daily Consumption of the Principal Nutrients

**Figure 6:**
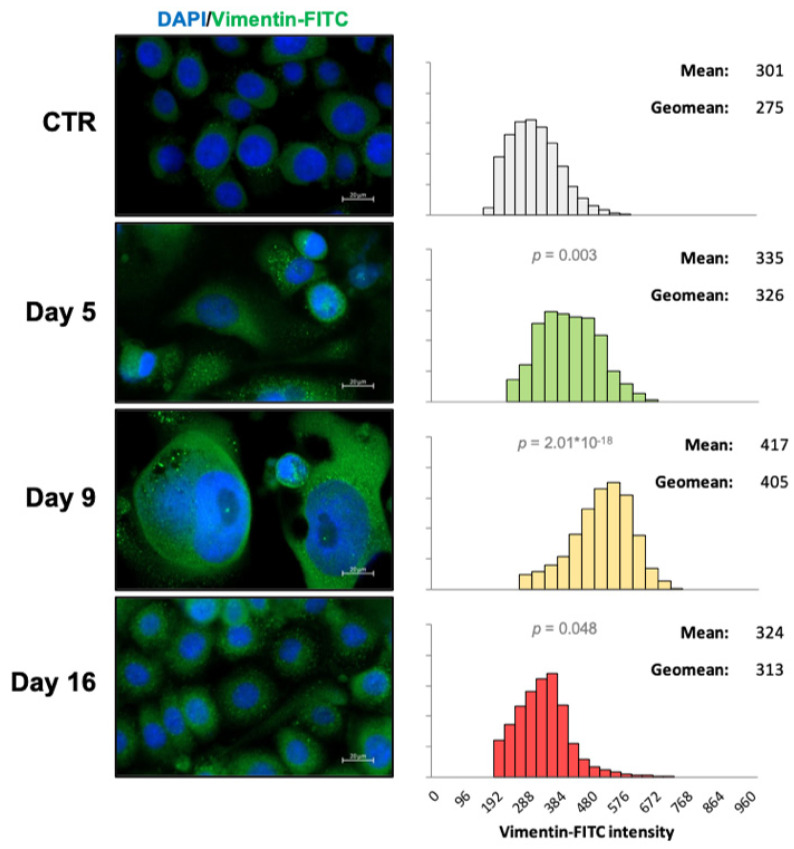
Dynamics of vimentin expression during the different phases of CRAC. Fluorescence microscope images and vimentin quantification (reported as the relative frequency distribution of fluorescence intensities) of PC3 cells after etoposide treatment at the indicated time points. The mean and geomean values are noted above each histogram. The images are representative of three independent experiments. Statistical significance (*p* < 0.05) of each distribution with respect to control cells (CTR) was calculated via the Mann–Whitney test.

Following exposure to etoposide, it was observed that cells underwent EMT, as indicated by the enhanced expression of the mesenchymal marker vimentin. Indeed, cells’ vimentin expression increased during the DE phase, reached a maximum in the QU phase, and declined during RE2. However, cells in this phase retained a higher level of vimentin than control cells (Figure 6).

Interestingly, PCA analysis allowed us to observe that cells in the QU phase, which express maximum vimentin, show an accelerated metabolic activity compared to all other phases (Figure 4A,B). In particular, the third PCA component, which explains 4% of the total variance, correlates with EMT, showing that among all the metabolites, QU-cells increased the consumption rate of principal nutrients and excretion of major end-products (Figure 4C,D).

**Figure 7:**
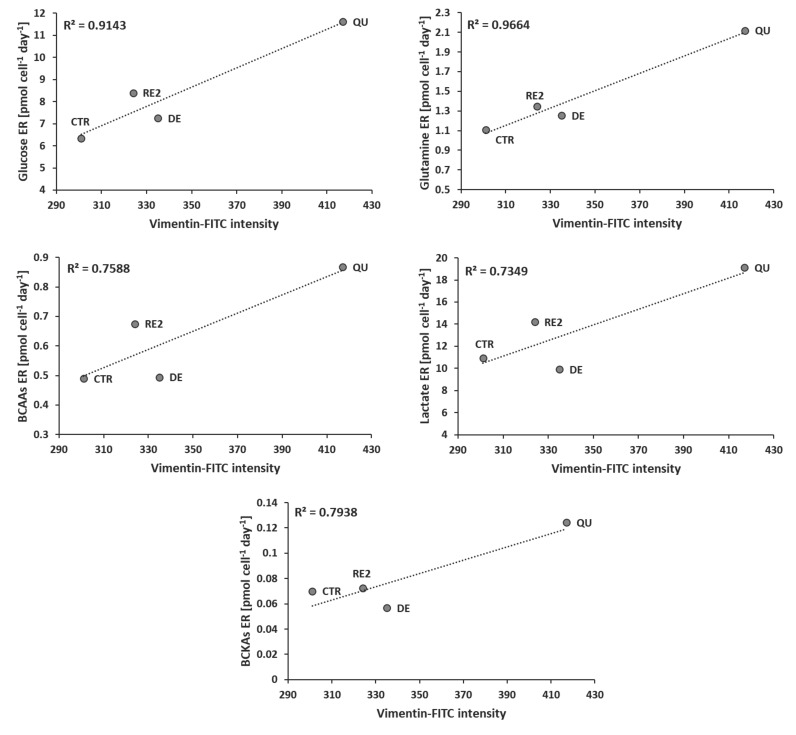
Vimentin expression after CTR, DE, QU, and RE2 phases vs. the *ERs* of the principal nutrients (glucose, glutamine, and BCAAs) and main excreted metabolites (lactate and BCKAs).

Figure 7 witnesses the close correlation between EMT, followed by the expression of vimentin, and the acceleration in the consumption of glucose, glutamine, BCAAs, and in the excretion of lactate and branched-chain keto acids (BCKAs), products of BCAAs metabolism.

### 3.6. The Recurrent Glycolysis/OXPHOS Switch of Cells after Etoposide Treatment

**Figure 8:**
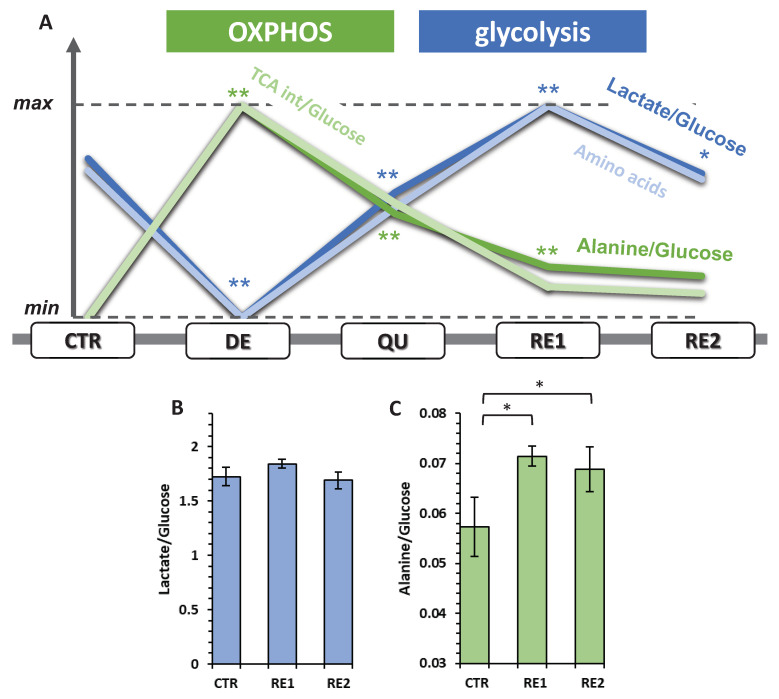
(**A**) Variation of the glycolysis and OXPHOS relative weight during CRAC. Significant lysine, threonine, methionine, phenylalanine, and tyrosine exchange rates. (**B**) Histogram of the Lactate/Glucose ratio for CTR, RE1, and RE2. (C) Histogram of the Alanine/Glucose ratio for CTR, RE1, and RE2. Significant differences are indicated with ** for *p* < 0.01 or * for 0.01 < *p* < 0.05.

Figure 8A shows the interplay between glycolysis and OXPHOS during the different phases that cells underwent after etoposide treatment. In this plot, lactate/glucose and alanine/glucose were used to measure the glycolytic and the OXPHOS efficiencies, respectively. During the DE phase, cells showed the minimum glycolytic activity and the maximum use of OXPHOS. Subsequently, these two energy pathways’ relative weights switched oppositely until glycolytic efficiency reached its maximum during RE1. Finally, comparing RE2 and CTR cells, we observed that the former showed a similar glycolytic activity (Figure 8B) but a higher OXPHOS capacity (Figure 8C) as if part of the rise during DE would have been retained. The changes observed for OXPHOS were closely tracked by changes in the *ERs* of TCA intermediates (succinate, citrate, fumarate, and α-ketoglutarate). The almost identical variation in the ratio of alanine/glucose and TCA intermediates/glucose reflects a close biochemical relationship between the amount of pyruvate converted to alanine from consumed glucose and the activity of the TCA cycle.

On the other hand, glycolysis activity variation during the phases was mirrored by the consumption of several essential amino acids (lysine, threonine, methionine, phenylalanine) and tyrosine. The fact that they are primarily essential allows us to hypothesize a close relationship between glycolytic activity and protein synthesis. This would explain why the glycolytic activity is higher for those cells that divide (CTR, RE1, and RE2) and therefore need to synthesize proteins.

With our data, the efficiency of glycolysis can be assessed by the lactate/glucose ratio, which can measure stoichiometrically how many glucose molecules are converted to lactate: a value of two reflects a total conversion between these two molecules and thus a highly efficient glycolytic activity. On the other hand, although the conversion of pyruvate to alanine has already been proposed as a marker of the level of OXPHOS [11], in this work, we corroborated this interpretation since the changes in alanine production (alanine/glucose ratio) traced back to the excretion of TCA intermediates, even though the only TCA intermediate formed from the conversion of pyruvate to alanine is α-KG.

## 4. Discussion

In this work, we have characterized the metabolic reprogramming of PC3 cells after etoposide treatment, after which cells undergo three subsequent phases: degenerative, quiescent, and repopulating. Not to perturb the cell-conditioned microenvironment, cells were never detached during the whole duration of the experiment, thus mimicking the in vivo context in which cells self-organize after the induced stress [7].

Comparison of the metabolism of untreated cells with those that survived an etoposide insult may generate hypotheses about biomarkers to predict prognosis and risk of tumor recurrence. In this context, metabolomics has been a particularly active field of study, allowing the evaluation of a profusion of candidates in different types of specimens [12]. Most of these studies have focused on discovering markers for diagnosis, which has allowed the identification of alanine, glycine, and sarcosine as good candidates [13]. However, in the design of these studies, the search for diagnostic markers has prevailed, and it has not yet been possible to detect candidates for prognosis and therapy prediction [12].

As a possible initial avenue in this direction, we have explored an exometabolomics approach to measure, through ^1^H-NMR spectroscopy, the concentration changes of 36 metabolites in the cell culture medium, which were then converted into exchange rates of 20 consumptions and 15 excretions. Only glycine showed a mixed behavior: it was consumed by rapidly proliferating cells, reflecting an evident demand from the exogenous source, and excreted by non-proliferating cells. Our results showed a close mathematical relationship between its exchange rate and the daily fold change in cell number (Figure 5). In a study of the exometabolome of 60 cancer cell lines, glycine and phosphocholine were the only metabolites among 111 to show a similar relationship with cell growth rate, which suggested the centrality of the glycine metabolism in the rapid proliferation of cancer cells [14]. This study also proved that exogenous glycine is incorporated by such cells for de novo biosynthesis of purines, the DNA nitrogen bases, and glutathione [14,15]. These two mechanisms can be considered the main contributions of glycine metabolism to the needs of fast-dividing cancer cells. Notably, glycine is a metabolite indicated in several metabolomics studies and was implicated as a marker for risk prediction, diagnostics, and even prognostics [12].

A surprising result regarded the *ERs* of lysine, threonine, methionine, phenylalanine, and tyrosine, which mirrored the glycolytic activity of these cells, measured as the lactate/glucose ratio. Most of these amino acids are essential, and their consumption is crucial to produce new proteins, an energy-demanding process, during the proliferative stage of a cell, as they represent about half of the total dry mass [16]. This strong correlation that we observed suggests glycolysis is the preferential way to support de novo protein synthesis. This hypothesis has already been raised by an integrated analysis in which the *ERs* have been used to characterize the metabolic behavior of 60 cancer cell lines. The authors hypothesized that the amount of ATP provided by aerobic glycolysis is proportional to the energy requirements for protein synthesis in cancer cells [15].

We then focused on the energetic reprogramming of PC3 cells during their reaction to the stress induced by etoposide exposure. Glucose represents the primary carbon source for PC3 cells in culture conditions and can be translated into energy by two main routes: aerobic glycolysis and OXPHOS. Normal prostate cells, specialized for citrate production, have high levels of intracellular Zn capable of inhibiting citrate isomerization in TCA. The consequence is a non-functional TCA and an energy balance favoring glycolysis over OXPHOS [17]. Instead, the malignant transformation of healthy prostate cells follows a unique energetic reprogramming. When these cells become tumoral, the Zn influx is inhibited, and the metabolic consequence is an increase in TCA activity and the OXPHOS/glycolysis balance [17].

**Figure 9:**
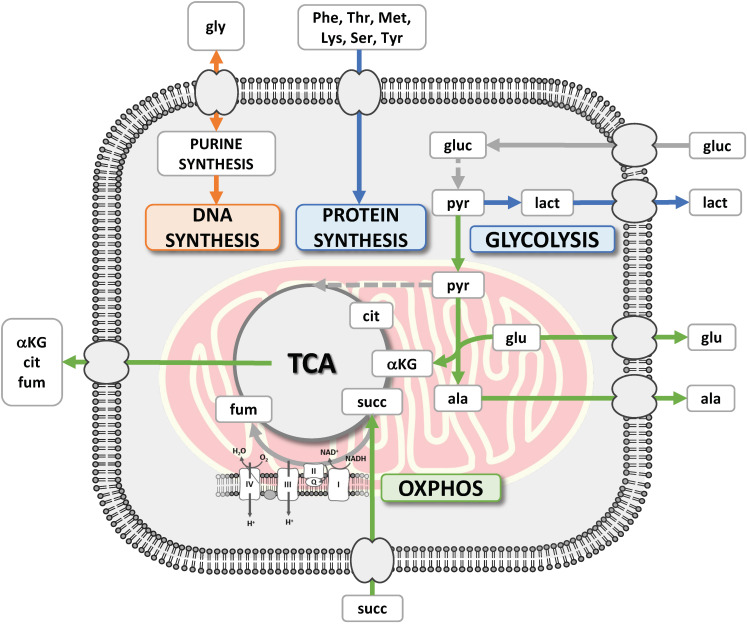
The consumptions and excretions delineate the metabolic routes to produce energy through glycolysis and OXPHOS or to support protein and DNA synthesis. The blue color for glycolysis and protein synthesis indicates their strong correlation.

All these three discussed features are summarized in Figure 9.

In our experiment, PC3 cells, as an immediate stress response in reaction to the drug, further increased the OXPHOS/glycolysis balance, as measured by the alanine production from glucose and the excretion of TCA intermediates. One possible hypothesis to explain this behavior is that many DNA-damaging anticancer agents enhance OXPHOS causing ROS production, mitochondrial dysfunction, and apoptosis [18]. In turn, glycolytic activity drops, being reduced to a minimum compared to the other phases studied, as generally occurs after severe stress [19] due to direct inhibition of glyceraldehyde-3-phosphate dehydrogenase (GAPDH) [20,21] and/or to the inhibition of two glycolytic limiting enzymes, phosphofructokinase (PFK) and pyruvate kinase (PK), by the caspases activated during apoptosis [22]. Afterward, the glycolytic activity resumes, reaching a maximum during RE1, while OXPHOS activity decreases; however, it does not go back to control values, maintaining a relatively high profile even during active cell proliferation.

Etoposide-treated PC3 cells, to resume proliferation, activate the pro-regenerative Phoenix Rising signaling cascade, which, in cancer cells, is not sufficient to support repopulation but requires profound epigenetic reprogramming to promote EMT [7]. This transition confers to tumor cells crucial properties like increased motility, invasiveness, and activity in degrading extracellular nutrients for invasion and metastasizing [23,24]. Noteworthy, our data strongly suggest, for the first time to our knowledge, that the dynamics of therapy-induced EMT correlates with cells’ energetic reprogramming. Indeed, we observed that cells undergoing EMT deeply remodeled their energetic metabolism, speeding up the *ERs* of the principal nutrients such as glucose, glutamine, and BCAAs, and the excretion products like lactate and BCKAs. In support of our results, it was previously reported that EMT increases glucose uptake and lactate excretion [25,26]. We also observed a high utilization of glutamine, probably related to an increase in glutaminase 1 (GLS1) activity, the inhibition of which has been observed to correlate with the disruption of EMT in endocrine tumors [27].

As mentioned, EMT could lead cells to consume faster BCAAs and excrete faster BCKAs than during the other phases. Leucine, isoleucine, and valine are amino acids necessary for protein synthesis, but they can also be catabolized to derive energy from hydrocarbon functional groups. Reversible transamination mediated by the enzymes BCAT1 and BCAT2 occurs in this process, producing glutamate and BCKAs. In the mitochondrion, dehydrogenated BCKAs form acetyl-coenzyme A and enter the TCA to generate ATP [28]. The faster consumption of BCAAs and excretion of BCKAs could therefore represent a new way for PC3 cells to meet the large energy demand required by the EMT program induced by the chemotherapeutic insult and could occur through the upregulation of BCAT1, which was reported to be a consequence of EMT in recurrent breast cancer cells [29].

The metabolic alternation between glycolysis and OXPHOS induced by the chemo-treatment also appears to correlate with the induction of the EMT program. Indeed, the glycolytic activity, initially suppressed by the immediate cell reaction to etoposide, resumes after activation of the EMT program, reaching a maximum during RE1, while OXPHOS activity decreases. This switch from OXPHOS to glycolysis, proposed as OGS [30], has recently been linked to EMT and seems to play an essential role in cancer development [26]. The rise in glycolysis can be explained by the activation of hexokinase 2, phosphofructokinase, and pyruvate kinase, which are reported as crucial in supporting EMT [31,32,33]. On the other hand, the decreased mitochondrial OXPHOS activity we detected can be associated with the downregulations of mitochondrial proteins already correlated with EMT and metastasis [34]. In endocrine tumors, the EMT process links with mutations in enzymes of the TCA cycle, like succinate dehydrogenase (SDH) and fumarate hydratase (FH) [26]. In the case of SDH, a mutation in the beta subunit is associated with an alteration in the use of glucose and glutamine, causing epigenetic modifications leading to EMT [35]. Mutation of FH is the first event that, in the end, leads to an increase in the level of fumarate, which acts as an oncometabolite increasing DNA hypermethylation, ROS production, and alteration in the mitochondrial structure [26]. In renal cancer, increased fumarate is one of the causes of EMT through inhibition of Tet dioxygenase-mediated demethylation of antimetastatic miR-200 [36].

However, contrary to the univocal relationship between cancer cell metabolism and mitochondrial dysfunction leading to the almost exclusive use of glycolysis [37], it has recently been observed that energy production through OXPHOS can be reprogrammed in tumor cells to cope with the high energy demand [38]. This has led to the possible existence of a hybrid glycolysis/OXPHOS phenotype to explain the metabolic plasticity of cancer cells, particularly regarding EMT, metastasis, and resistance to therapy [38,39]. Our results are further proof of this behavior; indeed, while the glycolytic activity of repopulating cells returns to control levels, they retain part of the higher mitochondrial activity developed in the degenerative phase. Respiratory capacity represents a crucial feature for invasion and progression [40], especially for sustaining malignant growth in prostate cancer cells [41], converting it into a potential prognostic marker and a therapeutic target. Remarkably, in the case of CRAC, this feature would also seem to support the adaptive capacity of the cells to the treatment and the subsequent regrowth (Figure 8A). Directly related to OXPHOS activity, the alanine/glucose ratio shows a significant increase in the QU and RP phases and remains significantly higher in repopulating cells than in untreated cells. Interestingly, alanine was one of the metabolic biomarkers indicated to determine progression from low-grade (LG) to high-grade (HG) PC [13].

## 5. Conclusions

Unlike other metabolomics studies, this work focus on a novel dynamic analysis of the metabolism of a cell line observed while reacting to chemotherapy treatment. This allowed highlighting the continuous switch of glycolysis and OXPHOS to cope with the consequences of the cytotoxic therapy, i.e., apoptosis, senescence, and reprogramming to resume proliferation. We observed that repopulating PC3 cells, just like the proliferating controls but unlike what happens during the non-proliferative phases, support protein biosynthesis, as shown by the speed with which they consume essential amino acids, taking energy from glycolysis. These are also the only phases when they consume glycine, an amino acid essential for purine biosynthesis and thus related to DNA synthesis. Notably, CRAC-promoted increased malignancy is marked by an increase in EMT and OXPHOS with respect to the untreated cells.

These results reinforce the concept that cancer cells need strong metabolic plasticity to face adverse events like chemotherapy. This feature can be acquired endogenously or stimulated by cytotoxic therapy, converting metabolic reprogramming into a new cancer hallmark [42]. In particular, the increased OXPHOS ability developed by PC3 surviving the etoposide treatment could represent a metabolic advantage to resist chemotherapy. From a clinical perspective, this knowledge could inspire future studies in detecting metabolic vulnerabilities to be used as targets for treating cancers, including advanced PC, that currently may rely only on palliative care.

## Data Availability

All data will be available upon request to the corresponding author. The data are not publicly available due to privacy.

## Supplementary Materials

The following supporting information can be downloaded at: https://www.mdpi.com/article/10.3390/metabo13010065/s1, Table S1: Growth rate constants for each phase, Table S2: *ER* values, Table S3: Percentage of carbon and nitrogen atoms for the principal nutrients.
